# Head and neck CT angiography to assess the internal carotid artery stealing pathway

**DOI:** 10.1186/s12883-020-01915-w

**Published:** 2020-09-03

**Authors:** Dongxu Wang, Zheng Li, Xiaoyang Zheng, Houyi Cong, Tianyu Zhang, Zhenghua Wang, Yuguang Wang, Jun He

**Affiliations:** 1 Departments of CT, The Second Affiliated Hospital of Qiqihar Medical College, 37 West Zhonghua Road, Qiqihar, Heilongjiang 161006 PR China; 2 Department of Electrophysiology, The Second Affiliated Hospital of Qiqihar Medical College, Qiqihar, 161006 Heilongjiang China; 3 Department of Anatomy, Qiqihar Medical College, Qiqihar, 161006 Heilongjiang China

**Keywords:** Computed tomography angiography, Stroke, Common carotid artery, Artery occlusive, Internal carotid artery, Steal blood

## Abstract

**Background:**

Common carotid artery occlusive disease (CCAOD) could form internal carotid artery steal pathways. Based on the diagnostic results of digital subtraction angiography (DSA), head and neck computed tomography angiography (CTA) was used to find the internal carotid artery stealing pathway after CCAOD.

Methods: The clinical and imaging data of 18 patients with CCAOD were retrospectively analyzed. DSA and CTA was used to evaluate internal carotid artery steal pathways.

**Results:**

Of the 18 patients with CCAOD, 10 patients found internal carotid artery steal pathways. There were 7 males and 3 females. Vascular ultrasound examination of all patients: The affected side had no blood flow in common carotid artery (CCA), and had retrograde blood flow in the external carotid artery (ECA). The blood flow of the affected side was decreased in the internal carotid artery (ICA), but it was antegrade. DSA diagnosed 10 cases of CCA occlusion and CTA diagnosed 10 cases of CCA occlusion. DSA and CTA found 6 internal carotid artery blood stealing pathways: ① Vertebral artery → occipital artery → external carotid artery → internal carotid artery (6 cases); ② Thyrocervical trunk → ascending cervical artery → occipital artery → external carotid artery → internal carotid artery (7 cases); ③ Costocervical trunk → deep cervical artery → occipital artery → external carotid artery → internal carotid artery (6 cases); ④ Affected side thyroid neck trunk → inferior thyroid artery → superior thyroid artery → external carotid artery → internal carotid artery (2 cases); ⑤ Contralateral external carotid artery → contralateral superior thyroid artery → affected superior thyroid artery → external carotid artery → neck Internal artery (2 cases); ⑥ Parathyroid neck → superficial cervical artery → occipital artery → external carotid artery → internal carotid artery (1 case).

**Conclusions:**

The patients with CCAOD can find the internal carotid artery blood stealing pathway through CTA.

## Background

Common carotid artery occlusive disease (CCAOD) refers to severe stenosis (stenosis ≥80%), sub occlusion (stenosis ≥95%) and occlusion caused by various causes, which can cause ischemic stroke [1]. Cerebral blood flow compensation is more complicated with CCAOD. When CCAOD occurs and the internal and external carotid arteries are unobstructed, the internal carotid artery pressure at the distal end of the lesion is significantly lower than that of the ipsilateral external carotid artery. The direction of blood flow in the artery is reversed, which is called “internal carotid artery steal” [2, 3]. The establishment of collateral circulation can relieve the symptoms of cerebral ischemia. The assessment of collateral circulation pathways in ischemic stroke is currently being actively studied. Due to the low incidence of CCAOD, few reports of blood stealing pathways have been reported.

There are many imaging methods for assessing internal carotid blood stealing in CCAOD [4]. Digital subtraction angiography (DSA) is the gold standard, but DSA is an invasive inspection method and expensive, and cannot be widely used for clinical screening [5]. Computed tomography angiography (CTA) is a non-invasive examination with high accuracy, and its consistency with DSA is 97% [6, 7]. Based on the diagnostic results of DSA, CTA was used to find the internal carotid artery theft pathway in CCAOD patients. Understanding blood stealing pathways is a prerequisite for personalized treatment of stroke patients.

## Methods

The study protocol was approved by the Regional Ethics Committee for Clinical Research of the Second Affiliated Hospital of Qiqihar Medical College. The study obtained informed consent from all patients. We retrospectively analyzed 4585 patients with ischemic stroke diagnosed in the Cerebral Vascular Stenosis Diagnosis and Intervention Center of the Second Affiliated Hospital of Qiqihar Medical College from December 2015 to December 2019. 18 cases were CCAOD, of which 10 cases had internal carotid blood stealing. There were 7 males and 3 females, aged 51 to 77 years, with an average of 63.6 years.

### Head and neck CTA imaging protocol

All patients were scanned with a 64-slice CT (Toshiba, Aquillion). The patient was supine with her/his head stopped and swallowing was prohibited during the scan. The scanning range was from the ascending aorta to the top of the skull. Using plain scan and enhanced scan schemes. The scanning parameters are as follows: The tube voltage was 120 kV, the tube current was 200 mA, the pitch was 41.0, and the scanning layer thickness was 0.5 mm; The enhanced scan parameters are as follows: The tube voltage was 120 kV, the tube current was 300 mA, the pitch was 41.0, and the scanning layer thickness was 0.5 mm. A double-barreled high-pressure syringe (USA, Mallinckrodt) was used to inject the contrast (Ultravist, 100 mL) 60 ~ 80 ml through the median vein of right elbow, and the injection rate was 5.0 mL / s. Immediately after the injection of contrast agent, 40 mL of saline was injected at a rate of 5.0 mL / s. The 5-6th cervical spine plane was used as the monitoring plane, and the left common carotid artery was monitored by ROI. The threshold was set to 120 HU, and scanning was triggered automatically after 8 s.

The CT image data was transmitted to a post-processing workstation. The two scanned images were deboned, subtracted, trimmed, and rotated. The image display method is demonstrated using volume rendering (VR), multi-planner reformation (MPR), maximum intensity projection (MIP), and curved planar reconstruction (CPR) .

Evaluation and Data Analysis.

All head and neck CTA images were evaluated by two experienced physicians, and the degree of vascular stenosis and collateral circulation pathways were judged. If the opinions were not consistent, a consensus decision was made after discussion.

### Neck vascular ultrasound examination

Neck vascular ultrasound was performed in all patients. A 4 MHz pulsed probe was used to detect the blood flow of both common carotid artery (CCA), internal carotid artery (ICA) and external carotid artery (ECA).

### DSA examination

All patients were examined by digital subtraction angiography (GE, Healthcare LCE) and high pressure syringe (Toshiba, Nemoto). In supine position, the patients were disinfected and covered with cloth in the conventional operation area. After local anesthesia, Seldinger puncture of femoral artery was performed, and angiography of aortic arch and vessels above aortic arch was performed respectively. The obtained image is processed by digitization as the gold standard of diagnosis.

## Results

**Fig. 1:**
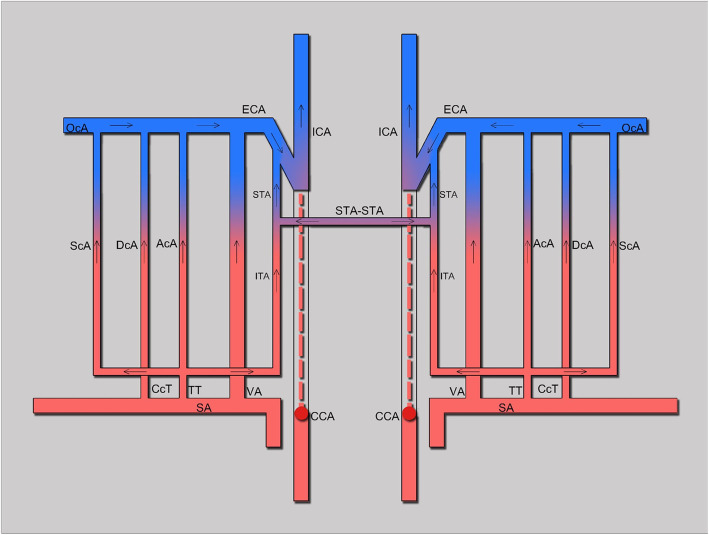
Schematic diagram of internal carotid artery blood stealing pathways. Abbreviations: CCA: Common carotid artery; ECA: External carotid artery; ICA: Internal carotid artery; SA: Subclavian artery; OcA: Occipital artery; VA: Vertebral artery; TT: Thyrocervical trunk; ACA: Ascending cervical artery; DCA: Deep cervical artery; SCA: Superficial cervical artery; CcT: Costocervical trunk; ITA: Inferior thyroid artery; STA: Superior thyroid artery

**Fig. 2:**
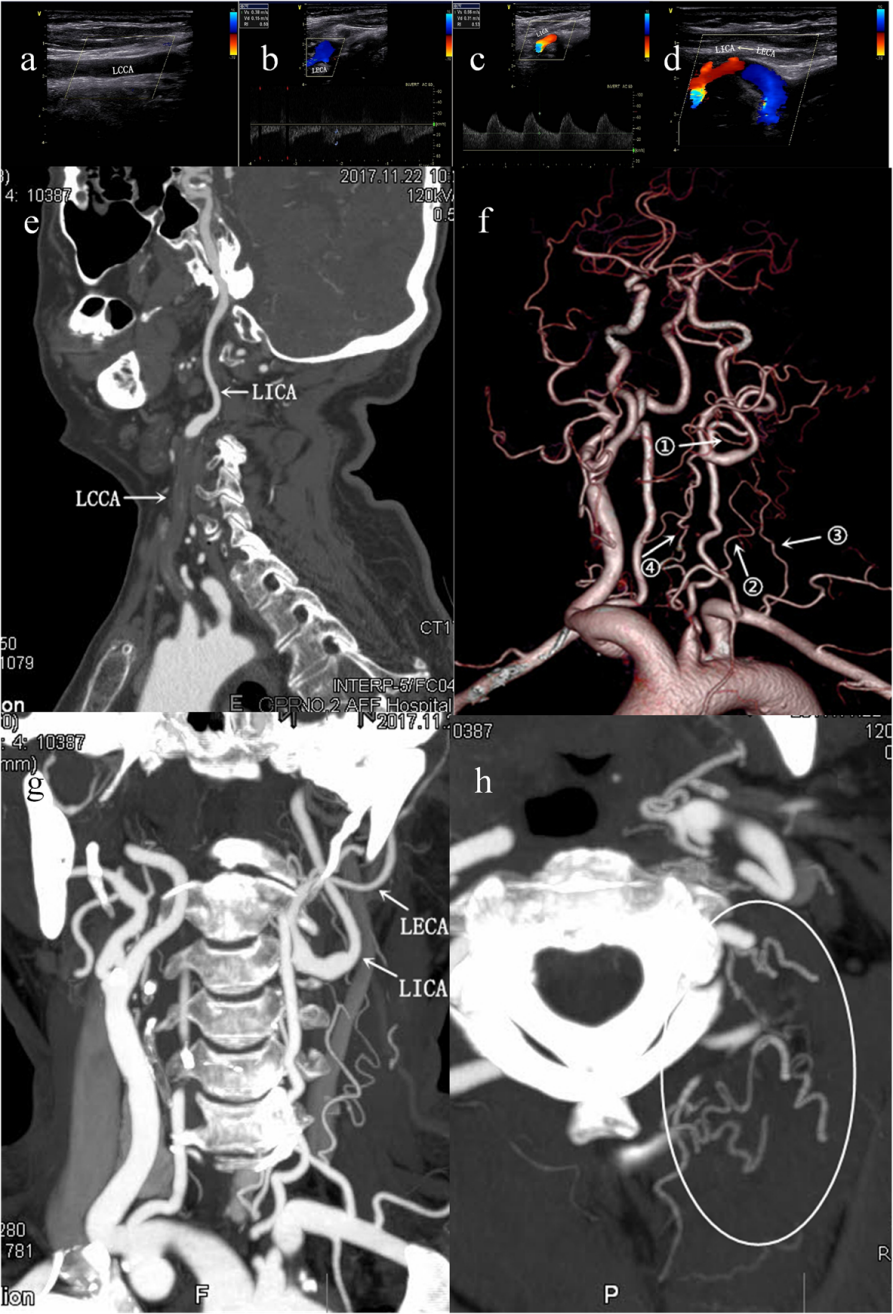
Patient 1, female. (Fig. 2a-d) Vascular ultrasound shows: **a** The left CCA had no blood flow, **b** the left ECA had retrograde blood flow, **c** the left ICA blood flow, the blood flow spectrum changes with low flow rate and low fluctuation, **d** the left ECA supplies blood to the left ICA. (Fig. 2e-h) CTA shows: **e** Curved Planar Reformation (CPR) shows: The left CCA occlusion, left ICA patency, **f** Volume Rendering (VR) shows: internal carotid artery stealing pathway, **g** Multi-planner reformation (MPR) shows: the left ICA steals blood access from left ECA, **h** Maximal intensity projection (MIP) shows: Deep cervical artery, ascending cervical artery- occipital artery forms the anastomotic vessels

**Fig. 3:**
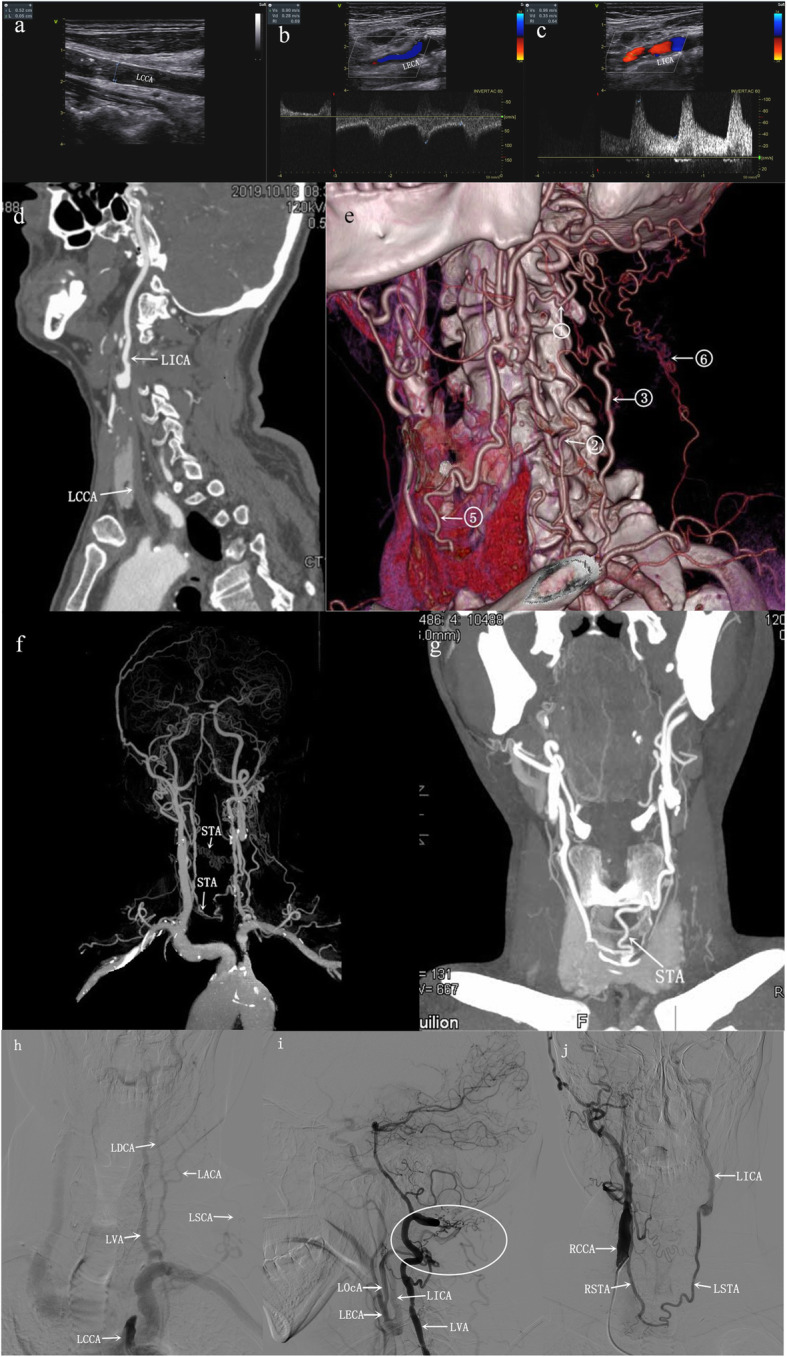
Patient 4, male. (Fig. 3a-c) Vascular ultrasound shows: **a** The left CCA had no blood flow, **b** the left ECA had retrograde blood flow, **c** left ICA blood flow shows low fluctuations in blood flow spectrum changes, (Fig. 3d-g) CTA shows: **d** CPR shows: The left CCA occlusion, left ICA patency, **e** VR shows: Internal carotid artery stealing pathway, **f** VR and **g** MIP shows: contralateral superior thyroid artery - affected superior thyroid artery forms an anastomotic blood vessel. (Fig. 3h-j) DSA showed: **h** Left superficial cervical artery positive image: the left CCA occlusion, the left ascending cervical artery (ACA), the deep cervical artery (DCA) and the superficial cervical artery (SCA) were thickened and lengthened. **i** Left vertebral artery lateral images display stealing pathway: left vertebral artery (VA), left ascending carotid artery (ACA), left deep carotid artery, left superficial carotid artery - left occipital artery (OcA) - left external carotid artery - left internal carotid artery . The circular area indicates that vertebral artery, deep carotid artery, ascending carotid artery and superficial carotid artery form anastomotic vessels. **j** Right common carotid artery anteroposterior images display stealing pathway: right common carotid artery - right superior thyroid artery (STA) - left superior thyroid artery - left external carotid artery - left internal carotid artery

18 CCAOD patients accounted for 0.39% of patients with ischemic stroke, of which 10 patients had internal carotid blood stealing (Table 1). Risk factors: There were 8 cases of hypertension, 7 cases of dyslipidemia, 3 cases of diabetes mellitus, 5 cases of smoking and 2 cases of drinking. Neck vascular ultrasound examination of all patients: The affected side had no blood flow in CCA, and had retrograde blood flow in the ECA. The blood flow of the affected side was decreased in the ICA, but it was antegrade. DSA diagnosed 10 cases of CCA occlusion and CTA diagnosed 10 cases of CCA occlusion. CTA and DSA found 6 internal carotid artery blood stealing pathways (Figs. 1, 2, and 3): ① Vertebral artery → occipital artery → external carotid artery → internal carotid artery (6 cases); ② Thyrocervical trunk → ascending cervical artery → occipital artery → external carotid artery → internal carotid artery (7 cases); ③ Costocervical trunk → deep cervical artery → occipital artery → external carotid artery → internal carotid artery (6 cases); ④ Affected side thyroid neck trunk → inferior thyroid artery → superior thyroid artery → external carotid artery → internal carotid artery (2 cases); ⑤ Contralateral external carotid artery → contralateral superior thyroid artery → affected superior thyroid artery → external carotid artery → neck Internal artery (2 cases); ⑥ Parathyroid neck → superficial cervical artery → occipital artery → external carotid artery → internal carotid artery (1 case).

**Table 1:** Demographics and blood stealing pathways of 10 patients

NO.	Sex	Atherosclerotic risk factors	Vascular ultrasound: Blood flow	CTA: Diagnosis and stealing pathway	DSA:Diagnosis and stealing pathway
1	Female	HT, DL	The left CCA had no blood flow, the left ECA had retrograde blood flow, and the left ICA blood flow decreased but was antegrade.	Left CCA occlusion,①②③④	Left CCA occlusion,①②③④
2	Male	Smoke, HT, DL, DM	The right CCA had no blood flow, the right ECA had retrograde blood flow, the right ICA blood flow was reduced but antegrade.	Right CCA occlusion, ①③④	Right CCA occlusion, ①③④
3	Female	HT	The left CCA had no blood flow, the left ECA had retrograde blood flow, and the left ICA blood flow decreased but was antegrade.	Left CCA occlusion, ①②③	Left CCA occlusion, ①②③
4	Male	Smoke, DL, DM	The left CCA had no blood flow, the left ECA had retrograde blood flow, and the left ICA blood flow decreased but was antegrade.	Left CCA occlusion, ①②③⑤⑥	Left CCA occlusion, ①②③⑤⑥
5	Male	HT, DL, DM	The left CCA had no blood flow, the left ECA had retrograde blood flow, and the left ICA blood flow decreased but was antegrade.	Left CCA occlusion, ①③	Left CCA occlusion, ①③
6	Male	Smoke, HT, DL	The left CCA had no blood flow, the left ECA had retrograde blood flow, and the left ICA blood flow decreased but was antegrade.	Left CCA occlusion, ②③	Left CCA occlusion, ②③
7	Male	Smoke, Drink	The right CCA had no blood flow, the right ECA had retrograde blood flow, the right ICA blood flow was reduced but antegrade.	Right CCA occlusion, ②	Right CCA occlusion, ②
8	Female	HT	The left CCA had no blood flow, the left ECA had retrograde blood flow, and the left ICA blood flow decreased but was antegrade.	Left CCA occlusion, ②	Left CCA occlusion, ②
9	Male	Smoke, HT, DL	The right CCA had no blood flow, the right ECA had retrograde blood flow, the right ICA blood flow was reduced but antegrade.	Right CCA occlusion, ①	Right CCA occlusion, ①
10	Male	HT, DL, Drink	The right CCA had no blood flow, the right ECA had retrograde blood flow, the right ICA blood flow was reduced but antegrade.	Right CCA occlusion, ②⑤	Right CCA occlusion, ②⑤

*Abbreviations*: *HT* Hypertension, *DL* Dyslipidemia, *DM* Diabetes mellitus

## Discussion

CCAOD was a rare clinical disease with clinical manifestations ranging from asymptomatic to severe symptoms of ischemic stroke [8]. The current incidence of CCAOD cannot be accurately assessed [9]. Seker.et al. reported that the prevalence of CCAOD was 0.34% [10], and other researchers reported that the incidence of CCAOD in patients with ischemic stroke ranges from 0.2 to 4% [11–13]. These data were consistent with the results of this article. The incidence of CCAOD in this article is 0.39%. In this paper, we found that 55.56% of CCAOD patients formed a carotid artery stealing pathway with DSA and CTA. The establishment of collateral circulation could guide the treatment and prognosis of stroke [14, 15].

In our study, men were more common (M / F = 2.33). Men were also more common in stroke patients [16]. The results of this article found that left common carotid artery occlusion was more likely to occur (60%), and we did not find patients with bilateral common carotid artery occlusion in our study. The risk factors in this article were similar to those in previous literature. Liebeskind report pointed out that hypertension, hyperlipidemia, diabetes, hypercoagulability and advanced age had a higher prevalence [17]. Our results showed that the prevalence of hypertension was 80% and the prevalence of hyperlipidemia was 70%. Some literature also reported rare risk factors, such as common carotid dissection, congenital dysplasia, etc.

When an artery in the body waed narrowed or occluded, the pressure at the distal end decreases. A siphonic effect could occur at this time, by “stealing” blood from adjacent vessels through the side branches of the arterial vessel. When the common carotid artery was narrowed or occluded, blood from the external carotid artery was stolen to the internal carotid artery, and we call it internal carotid artery stealing blood. Taking DSA as the gold standard, CTA had a high consistency with DSA in the diagnosis of CCA occlusion, and the coincidence rate was 100%. Analysis of CTA evaluation of internal carotid arty steaming pathways, 6 internal carotid arty steaming pathways were clearly displayed, the coincidence rate of CTA and DSA reached 100%, the results showed that CTA had a good evaluation effect on collateral circulation path. This is consistent with previous reports. Leng.et al. compared DSA and CTA images of CCAOD, and found that the total coincidence rate of CTA and DSA in judging the degree of vascular stenosis and displaying collateral circulation pathway was 100 and 98% [12]. DSA has higher spatial resolution, and can directly display collateral circulation and blood flow direction. However, DSA is an invasive examination method. In the process of head and neck angiography, it may lead to serious complications such as arterial dissection, vascular rupture, embolus shedding, etc. DSA is difficult to use in clinical work because of its high technical difficulty, high examination cost and large amount of contrast medium. In contrast, CTA is simple, convenient and inexpensive, and can avoid the occurrence of serious complications. Many literatures have proved that CTA and DSA have high consistency in the diagnosis of head and neck vascular stenosis and the display of collateral circulation pathways [16–18]. DSA can only perform angiography on a single vessel. On the basis of being familiar with the above six internal cardiovascular artery stabilizing pathways, it can avoid the omission of collateral circulation pathway during angiography.

Through DSA and CTA scan of the head and neck, we found 6 collateral circulation pathways for internal carotid stealing blood: ① Vertebral artery → occipital artery → external carotid artery → internal carotid artery; ② Thyrocervical trunk → ascending cervical artery → occipital artery → external carotid artery → internal carotid artery; ③ Costocervical trunk → deep cervical artery → occipital artery → external carotid artery → internal carotid artery; ④ Affected side thyroid neck trunk → inferior thyroid artery → superior thyroid artery → external carotid artery → internal carotid artery; ⑤ Contralateral external carotid artery → contralateral superior thyroid artery → affected superior thyroid artery → external carotid artery → neck Internal artery; ⑥ Parathyroid neck → superficial cervical artery → occipital artery → external carotid artery → internal carotid artery. Verbeeck.et al. reported the pathway ② through case reports [19], and Lai.et al. reported the pathway ③ through case reports [1]. CCAOD could provide blood perfusion to the diseased brain through 6 pathways. The results of this article showed that 60% of patients found pathway ①, 70% of patients found pathway ②, and 60% of patients found pathway ③. The occipital artery pathway was found in all patients, it can be deduced that the occipital artery pathway was the most important pathway for CCAOD to relieve cerebral blood perfusion. The results of this paper found that most internal carotid artery blood flow originated from the ipsilateral blood vessels. Only one case originated from the contralateral external carotid artery. So far, no related literature has reported six pathways of internal carotid blood stealing. DSA was the gold standard for diagnosing vascular disease. However, in clinical work, non-invasive vascular ultrasound and CTA tests were more practical [1]. The patients in this article were diagnosed with CCAOD and internal carotid artery steal by DSA. The display of collateral circulation pathways by neck vascular ultrasound was not as clear, intuitive, and accurate as CTA scans of the head and neck [20].

## Conclusions

The incidence of CCAOD is very low, and the blood stealing pathway is complicated. Common risk factors were hypertension and hyperlipidemia. We could use head and neck CTA to discover the internal carotid blood stealing pathway.

## Data Availability

The datasets used and analyzed during the present study are available from the corresponding author on reasonable request.

## Abbreviations

CCA: Common carotid artery
CCAOD: Common carotid artery occlusion disease
CTA: Computed tomography angiography
DSA: Digital subtraction angiography
ECA: External carotid artery
ICA: Internal carotid artery
SA: Subclavian artery
OcA: Occipital artery
VA: Vertebral artery
TT: Thyrocervical trunk
ACA: Ascending cervical artery
DCA: Deep cervical artery
SCA: Superficial cervical artery
CcT: Costocervical trunk
ITA: Inferior thyroid artery
STA: Superior thyroid artery
VR: Volume rendering
MPR: Multi-planner reformation
MIP: Maximum intensity projection
CPR: Curved planar reconstruction
